# Retraction Note: Topological analysis of carbon and boron nitride nanotubes

**DOI:** 10.1038/s41598-020-67084-5

**Published:** 2020-06-16

**Authors:** Awais Yousaf, Hanan Alolaiyan, Muhammad Nadeem, Abdul Razaq

**Affiliations:** 1 Department of Mathematics, The Islamia University of Bahawalpur, Bahawalpur, 63100 Pakistan; 2 Department of Mathematics, King Saud University, Riyadh, 11362 Kingdom of Saudi Arabia; 3 Department of Mathematics, University of Education Lahore, Jauharabad, 41200 Pakistan

Retraction of: *Scientific Reports* 10.1038/s41598-020-58372-1, published online 30 January 2020

The authors are retracting this Article.

After the publication of this Article it was brought to the authors’ attention that the results obtained for boron nitride are incorrect. Subsequently, the authors identified an error in Table 1, row 2, where the number of edges for (2,3) is incorrectly defined as 8(n-2). The correct formula is 8(n-1). As a result of this error, the results and conclusions reported for boron nitride are invalid.

All authors agree with the retraction.

